# The Impact of Different Pretreatment Processes (Freezing, Ultrasound and High Pressure) on the Sensory and Functional Properties of Black Garlic (*Allium sativum* L.)

**DOI:** 10.3390/molecules27206992

**Published:** 2022-10-18

**Authors:** Kai-Hui Chan, Chao-Kai Chang, Mohsen Gavahian, Bara Yudhistira, Shella Permatasari Santoso, Kuan-Chen Cheng, Chang-Wei Hsieh

**Affiliations:** 1 Department of Food Science and Biotechnology, National Chung Hsing University, 145 Xingda Rd., South Dist., Taichung City 40227, Taiwan; 2 Department of Food Science, National Pingtung University of Science and Technology, Pingtung 91201, Taiwan; 3 Department of Food Science and Technology, Sebelas Maret University, Surakarta City 57126, Indonesia; 4 Department of Chemical Engineering, Widya Mandala Surabaya Catholic University, Surabaya 60114, Indonesia; 5 Department of Chemical Engineering, National Taiwan University of Science and Techology, Daan Dist., Taipei 10607, Taiwan; 6 Institute of Biotechnology, National Taiwan University, Taipei 10617, Taiwan; 7 Graduate Institute of Food Science Technology, National Taiwan University, Taipei 10617, Taiwan; 8 Department of Optometry, Asia University, Taichung City 413305, Taiwan; 9 Department of Medical Research, China Medical University Hospital, Taichung City 404333, Taiwan

**Keywords:** black garlic, S-allyl-cysteine, sensory quality, polyphenol, freezing, high hydrostatic pressure

## Abstract

Black garlic (BG) is an emerging derivative of fresh garlic with enhanced nutritional properties. This study aimed to develop functional BG products with good consumer acceptance. To this end, BG was treated with freezing (F-BG), ultrasound (U-BG), and HHP (H-BG) to assess its sensory and functional properties. The results showed that F-BG and H-BG had higher S-allyl-cysteine (SAC), polyphenol, and flavonoid contents than BG. H-BG and F-BG displayed the best sensory quality after 18 days of aging, while 5-hydroxymethylfurfural (5-HMF), SAC, and polyphenols were identified as the most influential sensory parameters. Moreover, the F-BG and H-BG groups achieved optimal taste after 18 days, as opposed to untreated BG, which needed more than 24 days. Therefore, the proposed approaches significantly reduced the processing time while enhancing the physical, sensory, and functional properties of BG. In conclusion, freezing and HHP techniques may be considered promising pretreatments to develop BG products with good functional and sensory properties.

## 1. Introduction

Black garlic (BG) is an emerging functional food that has recently become popular in China and some other regions of the world [1], mainly because of its functional properties. BG can decrease the risk of developing diseases and contains various health-promoting phytoconstituents (e.g., phenolics, flavonoids, S-allyl cysteine, hydroxycinnamic acid derivatives), which may make it superior to fresh garlic [2]. Dietary supplementation with SAC has health benefits, as shown in some previous studies, including anti-inflammatory and anti-apoptosis effects [3]. Another study indicated that SAC has functional properties such as anti-neuroinflammatory, insulinotropic, antidiabetic, hepatoprotective, and cytoprotective effects [3].

For the traditional processing of BG, fresh garlic is cooked and incubated under high-temperature and high-humidity conditions [1], which not only changes the original pungent flavor but also provides greater functionality. Bae et al. [4] also indicated that after garlic was made into BG at 70 °C for 45 days, the main active substance in BG increased by 5.5-fold. The polysaccharides in garlic account for 75% of its dry weight. During the aging process, polysaccharides are decomposed into monosaccharides and disaccharides and increase to levels 10 times more than those of raw garlic, thereby leading to BG’s sweet flavor [5]. Phan et al. [6] noted that bound polyphenols can degrade into free polyphenols because of heat treatment during processing, which further improves the antioxidant properties and the levels of S-allyl-cysteine (SAC)—the main active substance in garlic. Heat treatment for 45 days at 40 °C resulted in an SAC concentration of 124.67 mg/g [4]. SAC can prevent indomethacin-induced gastric damage and increase the total antioxidant concentration, although the dose of SAC affects this preventive effect [3]. Moreover, researchers are exploring approaches that can further enhance the functional properties of BG. 5-Hydroxymethylfurfural (5-HMF), a furan compound with both aldehyde and alcohol functional groups, is present in many heat-treated foods, such as coffee and BG [7]. While some studies highlight the hazards of 5-HMF (e.g., carcinogenicity, liver and kidney toxicity, reducing glutathione content in cells, and DNA damage) [7], recent studies have revealed that 5-HMF has beneficial physiological activities (e.g., anti-hypoxic injury and improving blood circulation) and can act as an antioxidant and anti-allergen [2]. This compound is also an important intermediate product of the Maillard reaction [1], and its accumulation is closely related to the speed of garlic’s blackening during BG processing [8,9]. Therefore, the 5-HMF content can be used as an important monitoring indicator for the quality of BG.

Using different techniques to increase food safety and functionality has become a recent trend. Ultrasound and high-pressure processing have been reported, showing that tyrosinase activity be enhanced by ultrasound, further promoting the production of unique organoleptic substances, as in cocoa and fermented tea leaves [10,11]. High-pressure pretreatment can destroy intracellular structures, enhancing the Maillard reaction [8]. In our previous research, freezing, ultrasound, or high-pressure pretreatments were combined with low-temperature aging (40 °C for 6–9 days), which was found to be effective in modifying the biological structure of garlic, promoting the enzymatic activity, and increasing the functional ingredients by 4–10-fold [12,13]. The effects of processing methods on SAC compounds are generally only focused on the content of SAC [3]. However, further information on the effects of such pretreatments on the functional components and sensory attributes of the final product is needed. To date, many studies related to the combination of emerging technologies have been carried out to improve the characteristics of materials, to assist in processing, and to extend the shelf life of materials or products [14,15,16].

Therefore, the present study aimed to produce BG from fresh garlic samples pretreated by freezing, ultrasound, and high-pressure processing, as well as to assess the variations in SAC, total polyphenols, flavonoids, reducing sugars, and 5-HMF, along with sensory attributes. The pretreatment methods used were freezing, ultrasonic, and high hydrostatic pressure, because previous studies suggested that these methods can increase the SAC content in garlic. According to a previous study, garlic samples that were frozen in liquid nitrogen and stored at −80 °C had a greater concentration of SAC (1.03 mg/g) than untreated samples (0.85 mg/g) [13]. In comparison to raw garlic (0.85 mg/g), ultrasonic pretreatment at 28 kHz for 1 h can enhance the SAC concentration in garlic (1.07 mg/g) [13]. A recent study on high hydrostatic pressure pretreatment revealed that HHP increased SAC concentrations by about 7–10-fold, and the processing at 300 MPa for 15 min was reported to represent the best processing conditions for increasing SAC formation (from 0.51 to 5.60 mg/g) [12].

## 2. Results and Discussion

### 2.1. Effects of Different Pretreatments on Black Garlic’s Appearance, Reducing Sugar Contents, and pH Value

**Figure 1:**
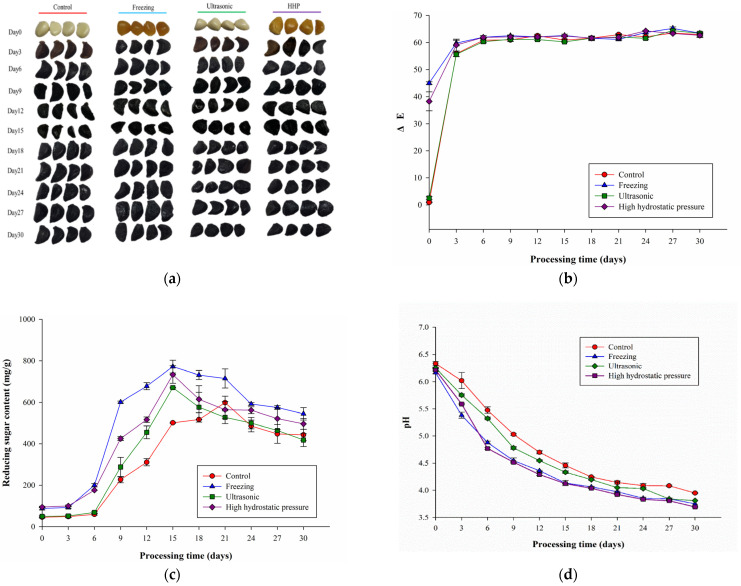
(**a**) Appearance, (**b**) ΔE, (**c**) reducing sugar content (mg/g dry weight), and (**d**) pH of garlic subjected to different pretreatment methods during thermal processing (70 °C, 80% RH for 30 days). All values are means ± standard deviations (*n* = 3). Control: only heat treatment at 40 °C, 80% RH for 6 days. Freezing: frozen at −20 °C for 30 h and then heat-treated at 40 °C with 80% RH for 6 days. Ultrasonic: 28 kHz for 2 h and then heat-treated at 40 °C with 80% RH for 6 days. High hydrostatic pressure: 300 MPa for 15 min and then heat-treated at 40 °C with 80% RH for 9 days.

The results for color change (ΔE) and appearance are shown in Figure 1a,b. On day 0 of the heat treatment, the degree of ΔE in the freezing and high-pressure pretreatment groups was higher than that in the ultrasonic and control groups. According to Che, Chen et al. [12], freezing and high-pressure pretreatment cause structural deformation and porosity in garlic, and enzymatic browning may be the cause of garlic browning caused by freezing and HHP pretreatments. Moreover, polyphenols and related oxidative enzymes such as polyphenol oxidase are bound to the cell wall and cell membrane. Once the plant structure is damaged, the substrate and enzymes are released, causing an enzymatic browning reaction. The ΔE of all groups increased rapidly on day 3 of heat treatment. In terms of the appearance, the color of the garlic was brown or black on the day 3. The reason for this is that under the environment of high temperature and high humidity, fructan is thermally cleaved to fructose and glucose, which react with amino acids to produce menerioids, which darken the garlic and lead to increased ∆E [8]. On days 6 and 9 of heat treatment, although the appearance of the garlic in all groups was black, the degree of ΔE varied with the severity of cell structure damage in the different pretreatments, ΔE in the freezing and high-pressure pretreatment groups was higher than that in the ultrasonic pretreatment group and the control group. The ΔE of all groups gradually leveled off after the 9th day of heat treatment, and the color was black.

According to previous studies, through microstructural analysis, it was found that freezing and high-pressure pretreatments have obvious destructive effects on garlic, causing structural deformation and porosity of the garlic [12,13]. Therefore, before the 12th day of heat treatment, the ΔE was higher than that of the ultrasonic pretreatment group and the control group, due to the severe degree of damage to the cell structure caused by the freezing and high-pressure pretreatments; after day 12, the ΔE of all groups plateaued.

The changes in reducing sugar contents in F-BG, U-BG, and H-BG aged for 30 days are shown in Figure 1c. It was found that after 15 days, the reducing sugar contents were highest, and those of the pretreated groups were higher than that of the control group (F-BG, 772.18 ± 30.85 mg/g dry weight; U-BG, 671.47 ± 1.89 mg/g dry weight; H-BG, 733.83 ± 42.31 mg/g dry weight; control, 501.46 ± 2.69 mg/g dry weight). Because garlic was aged under high temperatures after pretreatment, such conditions destroyed the cell structure, causing intracellular substances to flow, and decomposed more polysaccharides into monosaccharides and disaccharides, resulting in a higher reducing sugar content than that of the untreated group [9]. After 15 days, the reducing sugar contents of F-BG, U-BG, and H-BG began to decrease, indicating that as the aging time increased, the reducing sugar content decreased, which may have been because of the Maillard reaction [5]. Finally, the reducing sugar content was highest in the F-BG group (544.79 ± 29.92 mg/g dry weight), followed by the H-BG group (496.3 ± 26.26 mg/g dry weight). However, the control group reached the highest reducing sugar content after 21 days. This inflection point is called the reducing sugar balance point (RSBP), which may be positively associated with the improved processing efficiency of BG. The earlier equilibrium point indicated that the maturation time of BG was shorter, which could improve the processing efficiency. Before reaching the RSBP, the reducing sugar generation rate is faster than the consumption rate. After reaching the RSBP, the consumption rate of reducing sugars gradually becomes greater than the production rate [8].

As the processing time of BG increases, the pH value gradually decreases, and the increase in organic acid contents makes the taste of BG slightly sour [5,17]. The results of pH changes are shown in Figure 1d. During the heat treatment, the pH value of garlic gradually decreased. In addition to the release of organic acids due to the destruction of the garlic cell structure, organic acids were also generated during the heat treatment process, resulting in a decrease in the pH value of BG [5]. Liang et al. [18] noted that during the heating of garlic, the contents of acetic acid and formic acid increased significantly, mainly as a result of the cleavage of α-dicarbonyl and β-dicarbonyl of five-carbon or six-carbon sugars. During heat treatment at 70 °C, the pH values of the control, freezing, HPP, and ultrasonic pretreatment groups decreased from 6.33 to 3.95, 6.16 to 3.75, 6.21 to 3.69, and 6.25 to 3.81, respectively. Overall, the pH values of the pretreated samples were lower than that of the control group, indicating that the cell structure of these samples was damaged more severely, resulting in the release of more organic acids and lower pH values. Similarly, the results of Choi et al. [19] showed that the pH value of garlic after heat treatment at 70 °C for 28 days decreased from 6.33 to 4.07, similar to the trend of the control group in this experiment.

### 2.2. Effects of Different Pretreatments on Black Garlic SAC and 5-HMF Content

**Figure 2:**
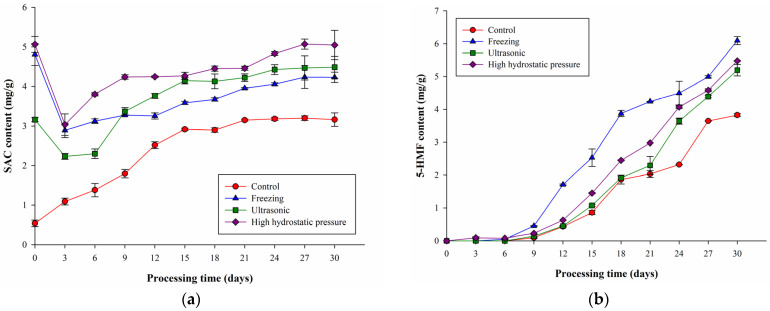
(**a**) SAC content (mg/g dry weight) and (**b**) 5-HMF content (mg/g) in garlic subjected to different pretreatment methods during thermal processing (70 °C, 80% RH for 30 days). All values are means ± standard deviations (*n* = 3). Control: only heat treatment at 40 °C, 80% RH for 6 days. Freezing: frozen at −20 °C for 30 h and then heat-treated at 40 °C with 80% RH for 6 days. Ultrasonic: 28 kHz for 2 h and then heat-treated at 40 °C with 80% RH for 6 days. High hydrostatic pressure: 300 MPa for 15 min and then heat-treated at 40 °C with 80% RH for 9 days.

Chen indicated that the SAC content in garlic could be increased 6-fold, 4-fold, and 10-fold through freezing pretreatment (−20 °C for 30 h), ultrasonic pretreatment (28 kHz for 2 h), and high hydrostatic pressure (HHP) pretreatment (300 MPa for 15 min), respectively [12,13]. Furthermore, we increased the SAC content in garlic via pretreatment and aged the BG under high-temperature and high-humidity conditions, observing the changes in SAC content under different pretreatment conditions. F-BG denotes BG subjected to freezing pretreatment, U-BG denotes BG subjected to ultrasound pretreatment, and H-BG denotes BG subjected to HHP pretreatment. The changes in SAC content in BG, F-BG, U-BG, and H-BG aged for 30 days are shown in Figure 2a. On day 0, the SAC content in all groups was similar to Chen’s findings [12,13]. During the aging period of F-BG, U-BG, and H-BG, there were no significant changes in the SAC contents of F-BG and H-BG because of the high-temperature conditions. It was speculated that during pretreatment γ-GTP had completely reacted with most of the GSAC, so that during the subsequent high-temperature and high-humidity aging stage the SAC formation tended to be flat. On day 3, the decreased SAC content may have been incurred by oxidation itself [20]. The literature indicates that γ-GTP will inactivate within 30 min at temperatures over 75 °C [4]; hence, SAC formation is less significant during the high-temperature aging stage of BG. Nevertheless, SAC formation in U-BG still gradually increased during aging—presumably because of the degree of cell disruption caused by ultrasound pretreatment—and the promotion of γ-GTP activity was lower than that of the freezing and HHP pretreatment methods, causing the GSAC and γ-GTP in the ultrasound pretreatment group to not fully react during the pretreatment period. In the subsequent aging stage, the cell walls were gradually destroyed because of the persistently high temperature, so the intracellular γ-GTP outflow contacting the GSAC gradually increased the SAC content. As a result, the SAC content in the U-BG group was higher than that in the F-BG and H-BG groups.

The final SAC contents of BG subjected to the three different pretreatment methods during the aging process were not significantly different (F-BG, 4.23 ± 0.13 mg/g; H-BG, 5.05 ± 0.37 mg/g; and U-BG, 4.49 ± 0.27 mg/g), but they were significantly higher than the SAC content of the control group (3.16 ± 0.17 mg/g). The main reason for this was that through the different pretreatment methods, the cell structure of garlic was destroyed, and SAC formation in the garlic was promoted [12,13]. The untreated group had an aging temperature (70 °C) that was higher than the optimal temperature of γ-GTP (40 °C). After prolonged exposure to high temperatures, the enzymes were gradually inactivated; thus, the SAC content of the control group did not change significantly after 12 days.

The results of changes in 5-HMF content are shown in Figure 2b. With the increase in the aging treatment time, the contents of 5-HMF in all groups showed an upward trend. On the ninth day of aging, the content of 5-HMF in the freezing pretreatment group began to increase most significantly, from 0.5 mg/g to 6.3 mg/g, followed by the HHP pretreatment group, from 0.3 mg/g to 5.8 mg/g. The 5-HMF content in the ultrasonic pretreatment group increased from 0.2 mg/g to 5 mg/g, while that in the control group increased from 0.2 mg/g to 3.8 mg/g.

Since the rate of 5-HMF formation is proportional to the rate of the Maillard reaction which, in turn, is proportional to the reducing sugar content. Li, Cao et al. and Li, Lu et al. [8,9] showed garlic with high-pressure pretreatment, the cell structure of black garlic is damaged, which accelerates the Maillard reaction rate, makes the rate of 5-HMF formation faster, and promotes the earlier aging of black garlic. It also can be seen from Figure 1 that compared with the other treatments, the freezing pretreatment accelerated the aging process of black garlic and was also the pretreatment group that produced the most reducing sugars. Therefore, this part of the results also showed that black garlic in the freezing pretreatment group produced more 5-HMF than the other pretreatment groups, probably because the damage to the garlic’s structure in the freezing pretreatment group was greater than that caused by the high-pressure and ultrasonic pretreatments.

### 2.3. Effects of Different Pretreatments on Black Garlic’s Polyphenol and Flavonoid Contents

**Figure 3:**
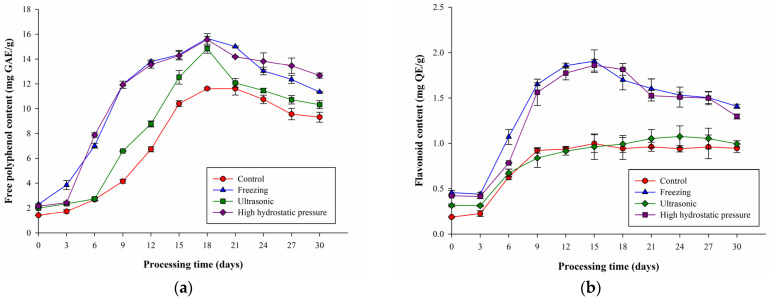
(**a**) Free polyphenol contents (mg GAE/g) and (**b**) flavonoid contents (mg QE/g dry weight) in garlic subjected to different pretreatment methods during thermal processing. All values are means ± standard deviations (*n* = 3).

The changes in total polyphenol contents in F-BG, U-BG, and H-BG aged for 30 days are shown in Figure 3a. The total polyphenol contents of F-BG, U-BG, and H-BG increased with time until reaching their highest values at 18 days (F-BG, 15.66 ± 0.15 mg GAE/g dry weight; U-BG, 14.84 ± 0.36 mg GAE/g dry weight; H-BG, 15.55 ± 0.49 mg GAE/g dry weight). There were no significant differences between these three groups. Afterwards, the polyphenol contents began to decline, but were still higher than that of the control group. This was related to the obvious destruction of the cell structures of garlic following pretreatment [12,13]; hence, more free polyphenols were formed, making the total polyphenol contents higher than that in the control group. Several reports have also confirmed that heat treatment can release polyphenols, increasing the free components of phenolic acids while reducing esterification, glycoside formation, ester binding, and formation of other phenol-containing macromolecular components, resulting in increased free phenols [19,21]. Moreover, the increase in polyphenol contents may have been due to the precursors of phenol molecules passing through phenolic non-enzymatic mutual conversion between molecules or to the release of polyphenols that are bound to the cell wall [22,23], whereas the decreased polyphenol contents and flattened rate after 18 days may have been due to the thermally sensitive polyphenols being easily degraded in high-temperature environments [24].

The changes in total flavonoid contents in F-BG, U-BG, and H-BG aged for 30 days are shown in Figure 3b. The flavonoid contents of F-BG and H-BG were significantly higher than those of the control and U-BG groups, and they increased with time until they reached their highest values after 15 days (F-BG, 1.91 ± 0.12 mg QE/g dry weight; H-BG, 1.86 ± 0.06 mg QE/g dry weight). The data revealed that the flavonoid contents of the F-BG and H-BG groups were two times higher than those of the control (1.00 ± 0.10 mg QE/g dry weight) and U-BG groups (0.96 ± 0.14 mg QE/g dry weight). The main reason for this phenomenon was that the freezing and high-pressure pretreatment methods caused a high degree of cell wall damage to garlic [12,13], and the contents of flavonoids in the F-BG and H-BG groups were significantly higher than those in the control and U-BG groups. Sukrasno et al. [25] noted that the endogenous bioconversion of flavonoid precursors or intermediates may have caused increased flavonoid contents—e.g., conversion of phenylpropane into flavones, or the polyphenol oxidase that was gradually inactivated at high temperatures—so the polyphenols and flavones were not oxidized but were accumulated in the samples. Among them, the total flavonoid contents in the F-BG, U-BG, and H-BG were higher than that in the control group. The reason for this phenomenon was that, in addition to the tissue damage caused by pretreatment, more flavones were released. Another reason may be that during the pretreatment period, the activity of chalcone synthase was promoted, which is a key enzyme for the production of flavonoids in plants [26], leading to the increased flavonoid content. The flavonoid content began to decrease after 15 days, presumably because of the thermal destruction of heat-sensitive components. Chaaban et al. [27] indicated that the reactivity of flavones to heat differs depending on their structures, and glycosylated flavones have better heat tolerance. Therefore, the rate of formation is less than the rate of degradation, resulting in reduced flavonoid content.

### 2.4. Principal Component Analysis of Functional Components of Black Garlic by Different Processes

**Figure 4:**
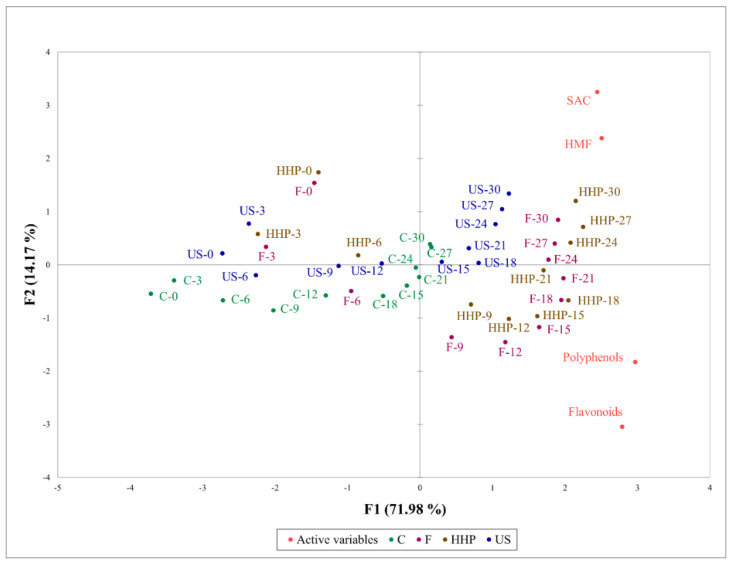
Principal component analysis of functional components of black garlic subjected to different processes.

A previous report used PCA to study the correlations between the variety, aging process, and functional properties or flavor of BG [28]. This study further explored the effects of different processing pretreatments on the functional components of BG during the ripening process. As shown in Figure 4, PC1 (F1) has 71.98% of the variance, PC2 (F2) has 14.17%, and the total is 82.16%.

The ultrasonic, freezing, and high hydrostatic pressure pretreatment groups all showed considerable influence on the generation of SAC and 5-HMF, but the effect of ultrasonic treatment on the generation of polyphenols and flavonoids was not as strong as that of the high hydrostatic pressure and freezing pretreatments. In our previous study, it was noted that ultrasound, HPP, and freezing can cause irreversible physical changes in the intercellular structure of garlic, further promoting the production of SAC, 5-HMF, polyphenols, and flavonoids [12,13]. This study also found that HPP and freezing had a more severe impact on the integrity of garlic cell nodules, so the use of these two pretreatment methods can affect the production of polyphenols and flavonoids more strongly than the ultrasonic pretreatment.

### 2.5. Correlation Analysis of Sensory Evaluation and Functional Components of Black Garlic by Different Processes

**Figure 5:**
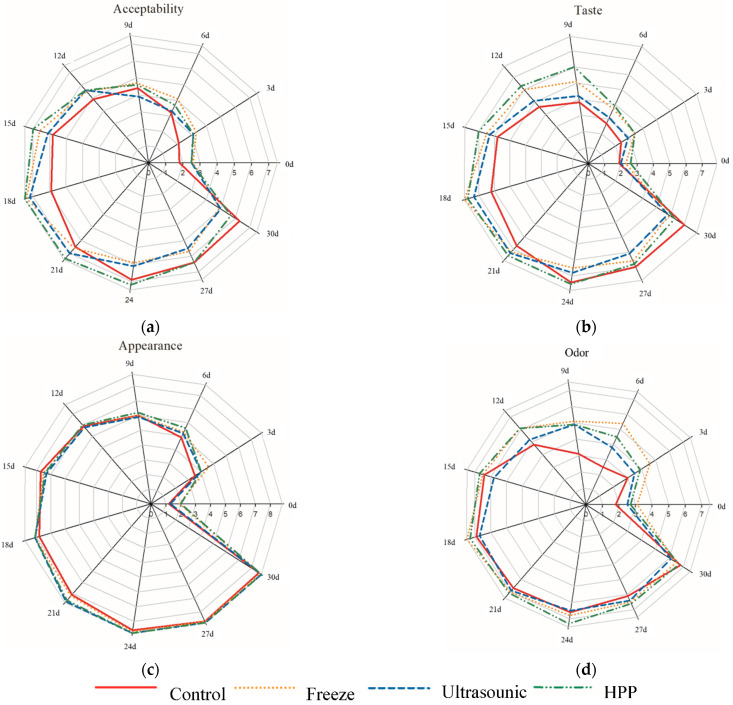
Effects of different processes on the sensory evaluation of black garlic: (**a**) acceptability; (**b**) taste; (**c**) appearance; (**d**) odor. *n* = 50.

According to Figure 5a, the HHP, ultrasonic, and freezing pretreatments can significantly improve the acceptability of BG. Although these pretreatments did not significantly improve the appearance of BG (Figure 5c), they did significantly change the taste (Figure 5b) and odor (Figure 5d) of BG.

Figure 5 also shows that the highest total scores for BG samples were recorded after 18 days for F-BG and H-BG, and there were no significant differences between 18 and 21 days for U-BG and 24 days for the control group. The literature indicates that BG has the best taste after 21–24 days [9], which is consistent with the observations from control group in the present study. Moreover, it was shown that BG under these conditions is acceptable to consumers, F-BG and H-BG having the best taste after 18 days of aging. Sensory evaluation and functional ingredients, including polyphenol and reducing sugar contents or the end of blackening of BG, have been the usual quality indicators [1,8].

To clarify Figure 5, the significance of the treatment in terms of sensory parameters is shown in Table 1. Based on Table 1, there was an increase in sensory acceptability scores over time (days), although each treatment had a different trend. In the control sample, on day 21, the panelists found that there were no significant differences in terms of sensory values up to day 30. However, for the freezing, ultrasound, and HPP samples, this trend started on days 24, 27, and 27, respectively. According to the results, all sensory parameters tended to increase with increasing aging time.

The relationships between functional ingredients and sensory evaluation were analyzed using Pearson’s correlation coefficient, as presented in Table 2.

According to the results, 5-HMF was highly correlated with acceptability, taste, appearance, and odor, with values of 0.627, 0.709, 0.770, and 0.732, respectively. In addition, SAC and polyphenols were also highly correlated with acceptability (0.637 and 0.550, respectively); other correlations included SAC and taste (0.567), polyphenols and taste (0.585), polyphenols and odor (0.647), and flavonoids and odor (0.598). These observations suggest that the functional components of BG have a relevant effect on consumer preferences, while 5-HMF and polyphenols have a more significant correlation with consumer preferences.

## 3. Materials and Methods

### 3.1. Materials

Garlic (*Allium sativum*) was harvested from Cihtong Township, Yunlin County, Taiwan and provided by Xiluo Agricultural Products Market Co., Ltd. Garlic weighing 30 ± 5 g was selected for the experimental samples, and 3 bulbs of garlic were used in each group of experiments, each with three replicates.

### 3.2. Sample Pretreatment

The garlic samples were divided into four groups: control, freezing, ultrasound, and high hydrostatic pressure (HHP) pretreatments. Samples were put in vacuum packages before the pretreatments. In the freezing pretreatment, the samples were frozen at −20 °C (Frt-U6009mfzw, Frigidaire Appliance Company, Charlotte, NC, USA) for 30 h. In the ultrasound pretreatment, the samples were processed at an ultrasonic frequency of 28 kHz (TST-TP, Taiwan Supercritical Technology Co., Ltd., Tainan, Taiwan) for 2 h. In the HHP pretreatment, the garlic samples were treated at 300 MPa (HPP600MPa/6.2L, Kuentai International Co., Ltd., Yunlin, Taiwan) for 15 min. After processing, the samples were removed from their packages. The freezing and HHP pretreatment groups were heat-treated at 40 °C with 80% relative humidity (RH) for 6 days, the ultrasonic group was heat-treated at 40 °C with 80% RH for 9 days, and the control samples were heat-treated at 40 °C with 80% RH for 6 days without any pretreatment for the generation of SAC. Then, the different processing groups and the control group were placed in a chamber at a constant temperature of 70 °C with 80%RH for 30 days to age the garlic into black garlic [12,13]. All of the abovementioned heat treatment procedures used a programmable constant temperature and humidity testing machine (CH-TH-5BP-A, E. Chung Machinery Company, Taoyuan, Taiwan). 

Sampling was performed every 3 days, and the BG samples were peeled and stored in a refrigerator at −20 °C to be analyzed later. For chemical and functional tests, samples were ground using a mortar and then homogeneously mixed. Then, 1 ± 0.5 g of BG mash was mixed with 10 mL of distilled water, processed with ultrasound for 30 min, and the extract was collected by gravity filtration.

### 3.3. Measurement of the Color Changes, Reducing Sugar Contents, and pH Value of Garlic

The color values of samples were measured using a colorimeter (NE-4000, Denshoku, Tokyo, Japan). The color of the samples was divided into *L*, *a*, and *b*, which represent white/black, red/green, and yellow/blue, respectively. Each full black garlic sample underwent five replications. Each individual black garlic bulb’s surface color was measured. Before taking the sample measurements, the equipment was calibrated against a reference white tile. Results were presented in the coordinate system of the CIELab tristimuli where *a* defines the red–green color range (*a* > 0 indicates redness; *a* < 0 suggests greenness), *b* describes the yellow–blue color range (*b* > 0 indicates yellowness; *b* < 0 indicates blueness), and *L* is a measure of brightness, from black (0) to white (100) [29]. The color difference (∆E) was calculated with Equation (1), where *L*0, *a*0, and *b*0 are the *L*, *a*, and *b* values, respectively, of fresh garlic and BG [5]:

(1) $$\Delta E=\sqrt{{\left(L1-L0\right)}^{2}+{\left(a1-a0\right)}^{2}+{\left(b1-b0\right)}^{2}}$$

The analysis of reducing sugars was performed using the phenol–sulfuric acid method [9]. Briefly, 1 mL extraction dilutions (diluted 1000 times) of BG samples from different treatment groups were poured into test tubes, and 0.6 mL of 3,5-dinitrosalicylic acid reagent (98%, Merck & Co., Inc., Kenilworth, NJ, USA) and 0.8 mL of 1 M NaOH (96%, Katayama Chemical Co., Ltd., Osaka, Japan) were added sequentially. The test tube was heated in boiling water for 15 min. After cooling, 5 mL of double-distilled water was detected by a microplate spectrophotometer (51119200, Thermo Fisher, Waltham, MA, USA) at 500 nm. Glucose (99%, Sigma-Aldrich, Burlington, MA, USA) solutions at different concentrations (0, 31.25, 62.5, 125, 250, 500, and 1000 μg/g) were used as standards. In the pH value analysis, 6 mL of the extraction solution was put into a test tube, the glass electrode of the pH meter (EL-20, Greifensee, Switzerland) was inserted into the test tube and dipped below the extraction solution, and the pH value was read [5].

### 3.4. Quantification of 5-HMF in Black Garlic by HPLC

We added BG puree (1 ± 0.5 g) to 10 mL of 80% methanol. After mixing, the mixture was sonicated for 30 min. The post-extraction solution was filtered through Whatman No. 4 filter paper and a 0.22 μm syringe filter. The filtered samples were analyzed by high-performance liquid chromatography (HPLC)-UV (L-7400, Hitachi, Hitachi shi, Japan). The column was a C18 column (250 mm × 4.6 mm ID, 5 μm, Nacalai Tesque Inc., Kyoto, Japan) with a flow rate of 1.0 mL/min. The mobile phase consisted of deionized water and acetonitrile (Merck & Co., Inc., Darmstadt, Germany) (88:12, *v*/*v*). The injected sample volume was 20 μL, and the detection wavelength was 284 nm [1].

### 3.5. Quantification of SAC in Garlic by HPLC

The BG samples were ground to a mash using a mortar and homogeneously mixed. Then, a fixed weight of BG mash (1 ± 0.5 g) was added to 10 mL of distilled water. After mixing, the mixture was processed with ultrasound for 30 min. The extraction solution was filtered through Whatman No. 4 filter paper and a 0.22 μm syringe filter. The filtered samples were subjected to HPLC with an ultraviolet detector (L-7400, Hitachi, Hitachi shi, Japan). The samples were separated on a C18 column (250 mm × 4.6 mm ID, 5 μm, Waters, Milfor, MA, USA) at a flow rate of 0.6 mL/min. Distilled water and acetonitrile (88:12, *v*/*v*) were used as the mobile phase. The sample injected volume was 20 μL, and the detection wavelength was 210 nm [30].

### 3.6. Measurement of Total Polyphenol and Flavonoid Contents

To assess the total phenolic content, the extract solution was diluted 100 times. Then, 40 μL of the test solution was taken and transferred into a microcentrifuge tube, and 400 μL of distilled water was added to it. Next, 20 μL of Folin–Ciocâlteu reagent (PanReac AppliChem, Hesse, Germany) was added to the test tube, and the tube was protected from light before adding 500 μL of 20% Na_2_CO_3_ (99%, Katayama Chemical Co., Ltd.) solution. After vortexing, the solutions were incubated in the dark for 20 min. The absorbance of the solution was measured at 750 nm. A standard curve was developed using various concentrations of gallic acid (99%, Scharlab, Barcelona, Spain) (0, 6.25, 12.5, 25, 50, and 100 μg/g) [31].

For analysis of flavonoid content, 0.1 mL of the diluted sample of the extraction solution was poured into a microcentrifuge tube, and then 0.3 mL of 99% ethanol (99%, Merck & Co., Inc., Darmstadt, Germany), 0.02 mL of 10% AlCl_3_ (97%, Katayama Chemical Co., Ltd.), 0.02 mL of 1 M CH_3_COOK (99%, PanReac AppliChem, Chicago, IL, USA), and 0.56 mL of deionized water were sequentially added. Thereafter, the solution was left to react at room temperature for 40 min. Absorbance was then detected at 415 nm. A standard curve was developed with quercetin, and the results were expressed as mg QE/g dw. The analyses were carried out in triplicate [6].

### 3.7. Principal Component Analysis (PCA)

The relationships between the different pretreatments (i.e., control, freezing, ultrasound and HHP) in term of the changes in the main functional components—i.e., SAC, 5-HMF, polyphenol, and flavonoid contents—during 30-day heat treatment of BG were analyzed by PCA. Sampling of BG was performed every three days, named C-Sampling Day, F-Sampling Day, US-Sampling Day, and HHP-Sampling Day. The PCA analysis was performed using a method described by Vidal, Manful [30] for Pearson’s correlation. XLSTAT software (Microsoft, Washington, DC, USA) was used to analyze these variables, and the significance level of the test was 5%.

### 3.8. Sensory Evaluation

Sensory evaluation was measured using the method previously described by Li, Lu et al. [9], with slight modifications. The BG samples pretreated using various methods were peeled. Sensory evaluation was conducted by 50 students and researchers with a food science background trained in the sensory evaluation course. The ratio of male to female respondents was 1:1, and the age distribution was 25–40 years. The appearance, taste, odor, and acceptability were evaluated and scored according to their preference. We used a nine-point scoring method to evaluate each feature, with a midpoint of 5, where 9 is the highest score and 1 is the lowest score.

### 3.9. Statistical Analysis

Data were expressed as means ± standard deviations. Statistical data processing was implemented through dispersion analysis using the SPSS 20 software. Statistical analysis was performed using one-way ANOVA and Duncan’s multiple range test, and statistical significance was set at *p* < 0.05. The XLSTAT 20 software was used to perform Pearson’s correlation coefficient analysis [19].

## 4. Conclusions

In this study, suitable processing technologies were established to enhance the quality of BG products. F-BG and H-BG had higher S-allyl-cysteine (SAC), polyphenol, and flavonoid contents than BG. Moreover, H-BG and F-BG displayed the best sensory quality after 18 days of aging, while 5-hydroxymethylfurfural (5-HMF), SAC, and polyphenols were identified as the most influential sensory parameters. Furthermore, the F-BG and H-BG groups optimal taste after 18 days, as opposed to untreated BG, which needed more than 24 d; therefore, the proposed approaches can significantly reduce the processing time while enhancing the physical, sensory, and functional properties of BG. In the future, researchers could produce BG products with high functionality and good sensory properties based on the findings of this study. Moreover, this technology could also be applied to the production of related fermented products to increase the biologically active components of functional foods. The emerging food processing technologies showed good potential in enhancing the product quality, while freezing and HHP techniques may be considered the most promising pretreatments to develop BG products with good functional and sensory properties.

## Figures and Tables

**Table 1:** Results of the sensory evaluation of black garlic based on a nine-point scoring method.

Treatment	Day	Acceptability	Taste	Odor	Appearance
Control	0	1.80 ± 0.63 ^e^	1.90 ± 0.57 ^g^	1.80 ± 1.23 ^d^	1.20 ± 0.63 ^h^
	3	2.10 ± 0.74 ^e^	2.40 ± 0.52 ^fg^	3.00 ± 1.15 ^c^	3.50 ± 1.08 ^g^
	6	3.20 ± 0.63 ^d^	2.70 ± 0.67 ^f^	2.50 ± 0.85 ^cd^	4.90 ± 1.20 ^f^
	9	4.40 ± 0.97 ^c^	3.80 ± 0.63 ^e^	3.10 ± 1.20 ^c^	6.00 ± 1.05 ^e^
	12	4.90 ± 0.57 ^c^	4.60 ± 1.07 ^d^	4.80 ± 1.03 ^b^	6.90 ± 1.29 ^d^
	15	5.80 ± 0.92 ^b^	5.80 ± 1.03 ^c^	6.40 ± 1.35 ^a^	7.70 ± 0.48 ^c^
	18	5.90 ± 1.10 ^b^	6.20 ± 0.92 ^bc^	6.90 ± 1.37 ^a^	7.80 ± 0.42 ^bc^
	21	6.50 ± 0.71 ^ab^	6.70 ± 0.82 ^ab^	6.70 ± 1.25 ^a^	8.10 ± 0.57 ^bc^
	24	6.90 ± 0.74 ^a^	7.40 ± 0.70 ^a^	6.60 ± 1.58 ^a^	8.60 ± 0.70 ^ab^
	27	6.40 ± 1.26 ^ab^	7.00 ± 1.15 ^a^	6.10 ± 1.52 ^a^	8.70 ± 0.48 ^a^
	30	6.30 ± 1.06 ^ab^	7.00 ± 0.47 ^a^	6.80 ± 0.92 ^a^	8.60 ± 0.70 ^ab^
Freezing	0	2.60 ± 1.17 ^g^	2.30 ± 0.95 ^f^	3.10 ± 1.10 ^e^	1.90 ± 0.74 ^g^
	3	3.30 ± 0.82 ^fg^	3.40 ± 1.26 ^e^	4.60 ± 0.52 ^d^	4.60 ± 1.96 ^f^
	6	4.10 ± 1.60 ^ef^	3.70 ± 1.25 ^e^	5.40 ± 1.07 ^cd^	5.30 ± 1.25 ^ef^
	9	4.70 ± 1.25 ^de^	5.10 ± 1.37 ^d^	5.10 ± 1.45 ^d^	6.00 ± 1.41 ^de^
	12	5.50 ± 1.08 ^bcd^	6.00 ± 1.33 ^cd^	6.10 ± 1.20 ^bc^	6.80 ± 0.79 ^cd^
	15	6.60 ± 0.70 ^ab^	6.50 ± 0.71 ^bc^	6.50 ± 0.97 ^ab^	7.50 ± 0.53 ^bc^
	18	7.40 ± 0.70 ^a^	7.90 ± 0.57 ^a^	7.50 ± 0.71 ^a^	8.10 ± 0.57 ^ab^
	21	6.60 ± 0.70 ^ab^	7.20 ± 0.63 ^ab^	7.00 ± 0.94 ^ab^	8.20 ± 0.63 ^ab^
	24	5.90 ± 0.88 ^bc^	6.50 ± 0.71 ^bc^	6.80 ± 1.23 ^ab^	8.70 ± 0.67 ^a^
	27	5.70 ± 1.06 ^bcd^	6.60 ± 0.84 ^bc^	6.50 ± 0.97 ^ab^	8.70 ± 0.67 ^a^
	30	4.90 ± 1.79 ^cde^	5.90 ± 1.52 ^cd^	6.50 ± 0.97 ^ab^	8.70 ± 0.67 ^a^
Ultrasound	0	2.50 ± 0.85 ^f^	2.00 ± 0.82 ^f^	2.50 ± 1.18 ^f^	1.30 ± 0.67 ^f^
	3	3.10 ± 0.88 ^ef^	2.90 ± 1.20 ^ef^	3.50 ± 0.53 ^e^	4.00 ± 1.70 ^e^
	6	3.30 ± 0.67 ^ef^	3.10 ± 0.57 ^e^	3.80 ± 0.79 ^e^	5.20 ± 1.23 ^d^
	9	3.90 ± 0.88 ^e^	4.20 ± 1.48 ^d^	4.90 ± 0.57 ^d^	5.90 ± 1.52 ^d^
	12	5.60 ± 1.07 ^cd^	5.10 ± 0.99 ^cd^	5.20 ± 0.92 ^cd^	6.80 ± 1.03 ^c^
	15	6.10 ± 0.88 ^bc^	6.30 ± 0.95 ^ab^	5.80 ± 0.79 ^bcd^	7.30 ± 0.67 ^bc^
	18	7.20 ± 0.63 ^a^	7.30 ± 0.95 ^a^	6.70 ± 0.82 ^ab^	8.10 ± 0.57 ^ab^
	21	7.00 ± 1.05 ^ab^	7.30 ± 0.95 ^a^	6.90 ± 1.10 ^a^	8.70 ± 0.48 ^a^
	24	6.10 ± 1.10 ^bc^	6.80 ± 1.03 ^ab^	6.50 ± 1.27 ^ab^	8.80 ± 0.42 ^a^
	27	5.50 ± 1.35 ^cd^	6.10 ± 1.29 ^bc^	6.40 ± 1.26 ^ab^	8.80 ± 0.42 ^a^
	30	5.00 ± 1.56 ^d^	5.80 ± 1.48 ^bc^	6.10 ± 1.20 ^ab^	8.80 ± 0.42 ^a^
HPP	0	2.50 ± 0.85 ^f^	2.60 ± 0.84 ^f^	2.70 ± 1.88 ^e^	2.00 ± 0.94 ^g^
	3	3.10 ± 0.74 ^ef^	3.40 ± 0.70 ^e^	3.90 ± 2.08 ^d^	4.00 ± 1.63 ^f^
	6	3.70 ± 1.06 ^e^	3.90 ± 1.10 ^e^	4.50 ± 2.07 ^cd^	5.60 ± 1.65 ^e^
	9	4.60 ± 1.07 ^d^	6.00 ± 1.05 ^d^	4.90 ± 0.53 ^c^	6.20 ± 1.69 ^de^
	12	5.60 ± 1.07 ^c^	6.30 ± 0.67 ^cd^	6.10 ± 1.45 ^b^	7.00 ± 1.05 ^cd^
	15	7.00 ± 0.94 ^ab^	7.00 ± 0.47 ^abc^	6.70 ± 1.15 ^ab^	7.40 ± 0.70 ^bc^
	18	7.50 ± 0.85 ^a^	7.70 ± 0.48 ^ab^	7.30 ± 1.32 ^a^	8.10 ± 0.57 ^ab^
	21	7.40 ± 0.70 ^a^	7.50 ± 0.71 ^ab^	7.10 ± 1.25 ^a^	8.60 ± 0.52 ^a^
	24	7.20 ± 0.92 ^ab^	7.50 ± 0.53 ^ab^	7.30 ± 1.10 ^a^	8.80 ± 0.42 ^a^
	27	6.40 ± 1.07 ^bc^	6.80 ± 0.42 ^bc^	6.60 ± 1.05 ^ab^	8.80 ± 0.42 ^a^
	30	5.70 ± 1.42 ^c^	6.30 ± 0.95 ^cd^	6.70 ± 1.03 ^ab^	8.80 ± 0.42 ^a^

Values are means ± SD (*n* = 3). In the same pretreatments, values with different superscripts within the same column are significantly different (*p* < 0.05).

**Table 2:** Correlation of quality indicators of black garlic.

Variables *	SAC	5-HMF	Polyphenols	Flavonoids	Acceptability	Taste	Appearance	Odor
SAC	**1**	0.539	0.692	0.618	0.637	0.567	−0.053	0.262
5-HMF	0.539	**1**	0.649	0.525	0.627	**0.709**	**0.775**	**0.732**
Polyphenols	0.692	0.649	**1**	**0.790**	0.550	0.585	−0.343	0.647
Flavonoids	0.618	0.525	**0.790**	**1**	0.412	0.491	−0.461	0.598
Acceptability	0.637	0.627	0.550	0.412	**1**	**0.888**	0.199	0.300
Taste	0.567	**0.709**	0.585	0.491	**0.888**	**1**	0.381	0.550
Appearance	−0.053	**0.775**	−0.343	−0.461	0.199	0.381	**1**	−0.005
Odor	0.262	**0.732**	0.647	0.598	0.300	0.550	−0.005	**1**

* Values in bold are different from 0 with a significance level alpha = 0.05.

## Data Availability

Not applicable.

## Sample Availability

Samples of the compounds are available from the authors.
